# Global characterization of the root transcriptome of a wild species of rice, *Oryza longistaminata*, by deep sequencing

**DOI:** 10.1186/1471-2164-11-705

**Published:** 2010-12-15

**Authors:** Haiyuan Yang, Liwei Hu, Thomas Hurek, Barbara Reinhold-Hurek

**Affiliations:** 1 Laboratory of General Microbiology, Faculty of Biology and Chemistry, University of Bremen, PO. Box 330440, D-28334 Bremen, Germany

## Abstract

**Background:**

*Oryza longistaminata*, an AA genome type (2 n = 24), originates from Africa and is closely related to Asian cultivated rice (*O. sativa L*.). It contains various valuable traits with respect to tolerance to biotic and abiotic stress, QTLs with agronomically important traits and high ability to use nitrogen efficiently (NUE). However, only limited genomic or transcriptomic data of *O. longistaminata *are currently available.

**Results:**

In this study we present the first comprehensive characterization of the *O. longistaminata *root transcriptome using 454 pyrosequencing. One sequencing run using a normalized cDNA library from *O. longistaminata *roots adapted to low N conditions generated 337,830 reads, which assembled into 41,189 contigs and 30,178 singletons. By similarity search against protein databases, putative functions were assigned to over 34,510 uni-ESTs. Comparison with ESTs derived from cultivated rice collections revealed expressed genes across different plant species, however 16.7% of the *O. longistaminata *ESTs had not been detected as expressed in *O. sativa*. Additionally, 15.7% had no significant similarity to known sequences. RT-PCR and Southern blot analyses confirmed the expression of selected novel transcripts in *O. longistaminata*.

**Conclusion:**

Our results show that one run using a Genome Sequencer FLX from 454 Life Science/Roche generates sufficient genomic information for adequate de novo assembly of a large number of transcripts in a wild rice species, *O. longistaminata*. The generated sequence data are publicly available and will facilitate gene discovery in *O. longistaminata *and rice functional genomic studies. The large number of abundant of novel ESTs suggests different metabolic activity in *O. longistaminata *roots in comparison to *O. sativa *roots.

## Background

Rice (*Oryza sativa *L.) is a staple food crop for about half of the world's population. In 2008, the total rice-harvested area and rough rice yield in the world were 155.7 million hectares and 661.8 million tons, respectively [International Rice Research Institute (IRRI) 2009]. However, the productivity of rice is severely affected by soil nitrogen nutrient deficiency worldwide. Commercially available urea fertilizer is the most widely used resource to meet a rice crop's nitrogen requirement, of which one third is lost through emission of greenhouse gasses and leaching, causing adverse environmental impacts [1-3]. To meet these challenges and develop environmentally sustainable rice production systems, much attention has been given to natural methods of biological nitrogen fixation (BNF) [4,5] or to increase nitrogen use efficiency (NUE) [6-8].

The genus *Oryza *comprises 24 species, including 2 cultivated (*O. sativa *and *O. glaberrima*) and 22 wild species with diverse ecological adaptation. These species are categorized into 10 recognizable genome types (AA, BB, CC, EE, FF, GG, BBCC, CCDD, HHJJ and HHKK) [9,10]. Wild rice has diversified over 40 million years. Wild species are tremendous gene reservoirs for domesticated rice improvement, as they possess many desirable traits, such as resistance to diseases and insect pests or tolerance to different kinds of stresses [11-14]. *Oryza longistaminata chev*. (2 n = 24, AA), broadly distributed throughout tropical Africa, is a perennial species with characteristics of long anthers, self-incompatibility, allogamy, strong rhizomes and high biomass production on poor soils. In spite of its overall inferior appearance, *O. longistaminata *has furnished genes for developing perennial rice [15,16] and for breeding blight disease resistance varieties [17]. To make better use of this potential, more genomic information is required, but there are only few batches of mRNAs or full-length cDNAs (FLcDNAs) of *O. longistaminata *in public databases, and no genome sequence is available.

Sequencing and analysis of expressed sequence tags (ESTs) has become a primary strategy for functional genomic studies in plants including novel gene discovery, gene expression profiling, microarray and molecular marker development, and accurate genome annotation. After completing the full genome sequence of *O. sativa *ssp. *japonica *cv. Nipponbare and the draft genome sequence of the *O. sativa *ssp. *indica *cv. 93-11 through a map-based sequencing strategy and through a whole-genome shotgun sequencing approach, respectively [18,19], much efforts were involved into rice ESTs projects. Approximately 1249,110 ESTs and >50,000 full-length cDNA sequences of cultivated rice are currently available in public databases. However, the genomic studies of rice wild relatives are still in their infancy with the exception of the generation of 5,211 leaf ESTs from the *O. minuta *(BBCC genome) and 1,888 leaf FLcDNAs from the *O. rufipogon *(AA genome) [20,21]. Especially roots are organs underrepresented in EST studies.

Therefore, a comprehensive survey of ESTs in roots of *O. longistaminata *was undertaken to provide an overview of *O. longistaminata *root transcriptome and thus a molecular basis for the identification of useful genes. As newly developed massively parallel 454 pyrosequencing allows rapid generation of sequence data and deep sequencing coverage with reducing labour and cost [22-24], we here characterized the first global root transcriptome of that wild rice species *O. longistaminata *by 454 GS-FLX pyrosequencing technology. This led to the discovery of a huge amount of novel ESTs which will facilitate gene mining and provide a basis for comparative studies within the genus *Oryza*.

## Results and Discussion

### Sequencing and assembly of 454 pyrosequencing ESTs

In order to obtain transcripts of genes that might be required for growth under nutrient stress, *O. longistaminata *plants were clonally propagated and were adapted to low-nitrogen conditions in unfertilized soil for several months. Mature plants with high biomass production (see Additional file 1) were subjected to RNA extraction from roots. As soil-grown roots often yield low quality RNA with inhibitory effects on enzyme activity (reverse transcription or PCR) [25], several RNA extraction methods were compared. A standard extraction protocol with Trizol yielded degraded RNA (not shown), while RNA extracted by a CTAB-based method was of high quality (Additional file 1).

Pooled RNA extracts from two extractions were used for normalization and sequencing of cDNAs. One GS-FLX 454 pyrosequencing run produced a total of 337,830 reads (87.3 Mb) with average sequence length of 258 bp (SD = 24, range = 60-925) from root cDNAs of *O. longistaminata*. After removal of adaptor sequences, polyA tail and low quality sequences, 337,471 reads remained with a total length of 66.7 Mb and an average length of 197 ± 61 bases, ranging from 20 bp to 393 bp (Additional file 2). Only sequences above 100 bp of length were further considered. Clustering and assembling of these sequences produced 43,423 contigs and 32,708 singletons. These data were trimmed again by removing those showing homology (E-value cutoff, e^-5^) to sequences of bacteria, fungi or metazoa, resulting in a total of 71,367 processed unique sequences. The length of contigs varied from 101 bp to 2082 bp with an average of 299 bp, and that of singlets ranged from 101 bp to 393 bp with an average of 215 bp (Additional file 2).

The majority of reads was in length of 201-300 bp (95% out of raw reads), which was consistent with the 454 GS-FLX sequencing capacity. The size distribution of *O. longistaminata *consensuses after assembly was shown in Table 1, revealing that 92% of them fell between 100 and 500 bp in length. Although none of singlets was longer than 500 bp in the whole EST dataset, there were 3,277 contigs with sequence length larger than 500 bp (Table 1). In addition, 21,762 contigs (53%) were still less than 250 bp. This might be due to the short length of the sequencing read and/or the low coverage of the transcriptome represented in this dataset. Most of the contigs were derived from few reads. 11,949 (29% out of contigs) and 7,226 (17.5% out of contigs) consensuses were derived from 2 and 3 reads, respectively (Additional file 3).

**Table 1:** Size distribution of Oryza longistaminata ESTs after assembly

	Contigs	Singletons	Total
101-250	21762 (53%)	26977 (89%)	48739 (68%)
251-500	16150 (39%)	3201 (11%)	19351 (27%)
501-750	2663 (6%)		2663 (4%)
751-1000	468 (1%)		468 (< 1%)
> 1000 bp	146 (< 1%)		146 (< 1%)

Total number	41189 (100%)	30178 (100%)	71367 (100%)
G+C	42.7%	41.7%	42.3%
Average length (bp)	299	215	263
Maximum length (bp)	2082	393	
Average reads	6.4		

The sequence data obtained were in a similar range as for other plant EST sequencing projects using this technology [26,27], however with a slightly higher read length, demonstrating the power of this approach to deliver large EST datasets.

### Mapping ESTs to the *O. sativa *genome and transcriptome revealed novel ESTs

**Figure 1:**
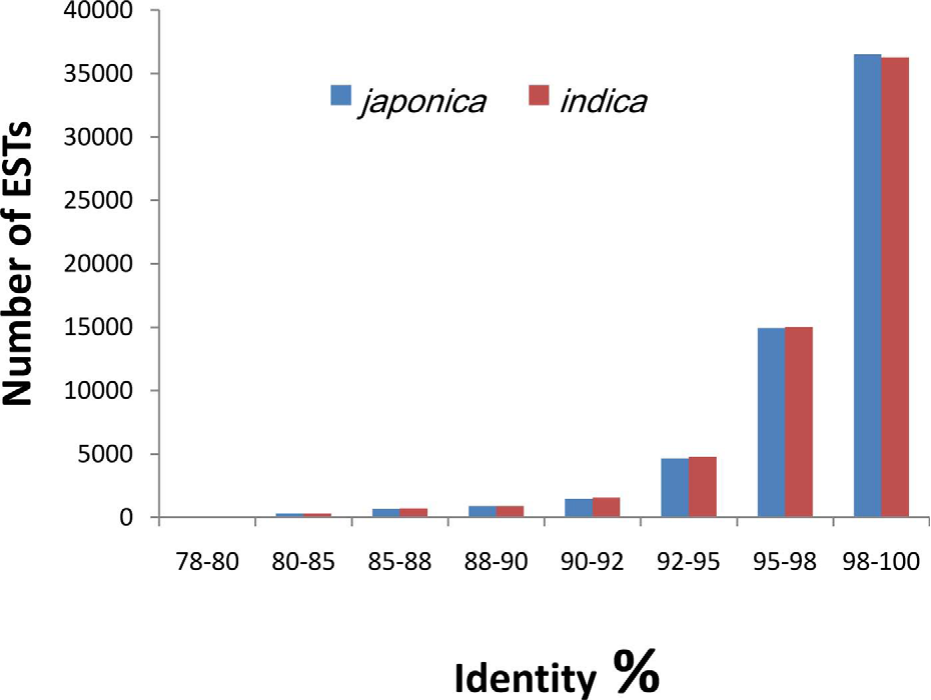
**Similarity distribution (percent of DNA identity) of *O. longistaminata *ESTs showing homology with cultivated rice**.

Comparison of the unique EST sequences of *O. longistaminata *to chromosomal and expressed sequences of *O. sativa *revealed a large set of two types of novel ESTs, those previously not found to be expressed by rice, and those not even detected in the rice genome. The ESTs were aligned to genomic sequences of two *O. sativa *varieties, *japonica *- type Nipponbare http://rgp.dna.affrc.go.jp/IRGSP/ and *indica *-type 93-11 http://rice.genomics.org.cn/rice/index2.jsp, by using the BLASTN program with an E-value cut-off of e^-5^. In total, 60,155 (84.3%) out of 71,367 sequence tags were anchored in rice genome. These sequences mapped on all the 12 rice chromosomes (Table 2) with almost equal distribution, chromosome 1, 2, 3 harbouring large amount of EST sites accounting for approximately 40% of a total of 60,155 EST sites. The distribution corresponds well to the size of the chromosomes, which highlights the close relationship between these two species. These anchored ESTs also had a relatively high sequence identity with *O. sativa *sequences ranging from 78% to 100%, with an average of 97%. The sequence identity distribution of 60,155 *O. longistaminata *ESTs is shown in Figure 1, revealing that 61% of them had a sequence similarity higher than 98%.

**Table 2:** Distribution of the consensus sequences in rice genome

**Chromosome^a^**	***japonica***		***indica***	
	
	**NO. of ESTs**	**Percentage**	**No. of ESTs**	**Percentage**
	
1 (43.26 Mb)	8194	13.7	8022	13.4
2 (35.93 Mb)	7114	11.9	7121	11.9
3 (36.41 Mb)	7773	13.0	7714	12.9
4 (35.28 Mb)	4959	8.3	4915	8.2
5 (29.89 Mb)	4867	8.2	4891	8.2
6 (31.25 Mb)	4947	8.3	4827	8.1
7 (29.70 Mb)	4486	7.5	4300	7.2
8 (28.44 Mb)	4248	7.1	3901	6.5
9 (23.01 Mb)	3186	5.3	2989	5.0
10 (23.13 Mb)	3136	5.3	2879	4.8
11 (28.51 Mb)	3250	5.4	2539	4.3
12 (27.50 Mb)	3487	5.8	2934	4.9
Unknown^b^			2684	4.5
Total	59647	100	59716	100

^a^The length of 12 Nipponbare (*japonica *cv.) chromosomes is indicated.

^b ^Genes not assembled into chromosomes of cv. 93-11.

**Figure 2:**
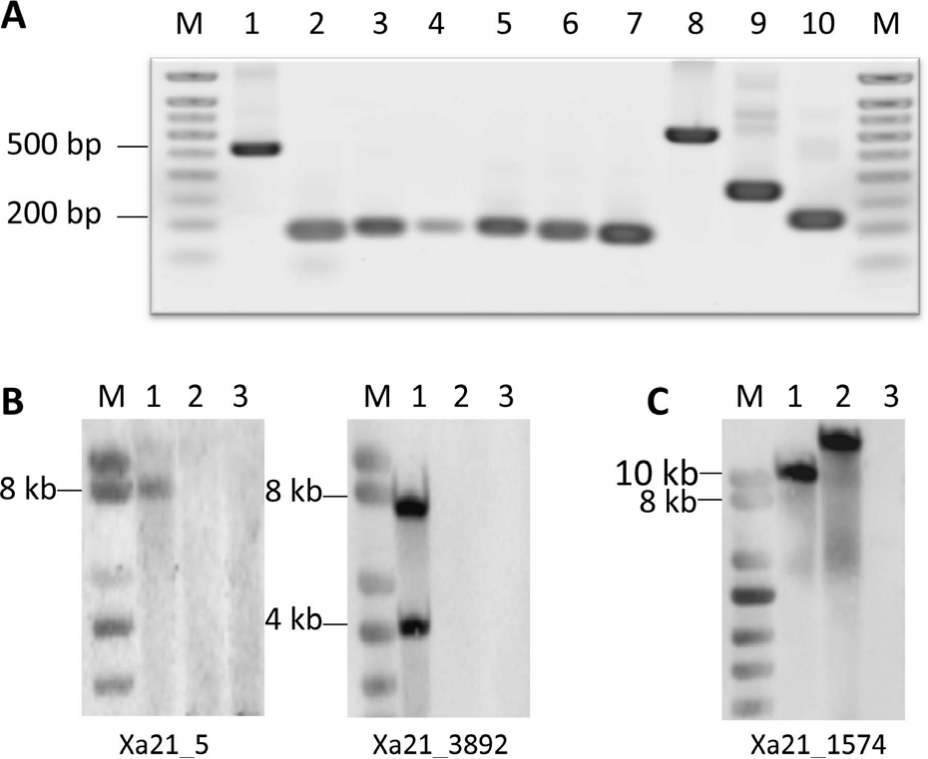
**Anaysis of selected novel transcripts by reverse transcritption (RT)-PCR and Southern blot analysis**. A. Expression of selected ESTs in *O. longistaminata *roots. M: DNA marker; 1: Xa21_5; 2: Xa21_11*; 3: Xa21_18; 4: Xa21_140*; 5: Xa21_202*; 6: Xa21_289; 7: Xa21_1050*; 8: Xa21_3892; 9: Xa21_20888; 10: Xa21_26755*. * indicated that those ESTs could also be amplified from root cDNAs of cultured wild rice seeds. B. Southern blotting analysis of novel ESTs. M: DNA marker; 1: O. longistaminata genomic DNA; 2: IR36 genomic DNA; 3: Nipponbare genomic DNA.

Among 60,155 ESTs, 508 could only be aligned to *indica *genome sequences and 439 could specifically match *japonica *genome sequences (see Additional file 4). Among these ESTs, 164 out of 508 or 247 out of 439 had high similarity (cut-off score above 100) to *indica *or *japonica *cDNA/ESTs sequences, respectively. They might be *indica*-specific or *japonica*-specific genes, or they may map to gaps in the rice genome sequences. The latter assumption may be likely for part of these ESTs, as 74 out of the 164 *indica*-specific genes mapped to ESTs detected in *japonica *with highest score, and 22 out of 247 *japonica-*specific ones to *indica *ESTs. One of the 508 ESTs, Xa21_1574, was selected for further analysis. The Southern blot analysis was consistent with the BLAST results (Figure 2). Our findings indicated that *O. longistaminata *had parallel similarity to *japonica *and to *indica *rice at the DNA level.

In order to assess how many *O. longistaminata *ESTs had already been detected as expressed genes in *O. sativa*, the ESTs mapping onto the *O. sativa *genomes were also compared with the Knowledge-based Oryza Molecular Biological Encyclopedia (KOME, http://cdna01.dna.affrc.go.jp/cDNA/) cDNA collection, the *indica *cDNA database http://www.ncgr.ac.cn/ricd/, and the NCBI rice EST database. 83.3% matched to *O. sativa *genes found to be expressed previously.

A large amount of ESTs (9,993 or 16.7%) had previously not been detected as expressed. For most of them, we did not find homologies to predicted gene models: Inspection of the 30 longest ESTs showed that 67% shared sequence similarity with *O. sativa *but not to predicted genes, 23% with genes of predicted functions, and 10% with genes encoding hypothetical proteins. This was also reflected in the lack of functional assignments (see below), as after *in silico *translation only for a small fraction (777) of these ESTs could be assigned according to Gene Ontology (GO). This emphasizes the power of the next generation sequencing approach to detect novel transcripts or even novel genes. As the *O. sativa *genome may still contain regions that are not fully annotated, our ESTs might indicate as yet unpredicted genes or UTRs that might be functional in *O. sativa *as well. On the other hand, *O. longistaminata *might express a special set of genes in comparison to *O. sativa*, due the particular conditions - being adapted to low availability of external nitrogen sources-, or due to the interspecies differences in expression.

As another category of novel ESTs, in total, 11,212 (15.7%) of 71,367 unique EST sequences could not be mapped to the *O. sativa *chromosomes by homology search against genomic sequences. Among them, 250 matched the publicly available *O. sativa *mRNAs or ESTs. The remaining 10,962 sequence tags showed no significant sequence identity (cut- off e^-5^) with any rice genomic or expressed sequences in public database. Among these, only a very small number (740) had a significant hit in NCBI non-redundant (NR) nucleotide database or ESTs database. The remaining 10,222 ESTs may therefore represent novel genetic material present in *O. longistaminata *and other root-residing eukaryotes.

### Functional classification of *O. longistaminata *ESTs

The consensus sequences were annotated for sequence similarities using the BLASTX translated sequence comparison against the NCBI non-redundant (NR) protein database. Among the 71,367 contigs and singlets, 34,510 (48.4%) had at least a significant alignment to exisiting gene models in the NR database at an E-value cut-off of e^-5^. A majority (51.6%) of the *O. longistaminata *sequences did not match any known protein sequences. Most of the 10,962 novel sequence tags (15.4%) fell into this category. This can partly be attributed to the short length of most of these uni-ESTs, or a large fraction of the ESTs might represent untranslated regions. Mapping those uni-ETSs to rice gene models supported this assumption. https://www.gabipd.org/database/cgi-bin/GreenCards.pl.cgi.

**Figure 3:**
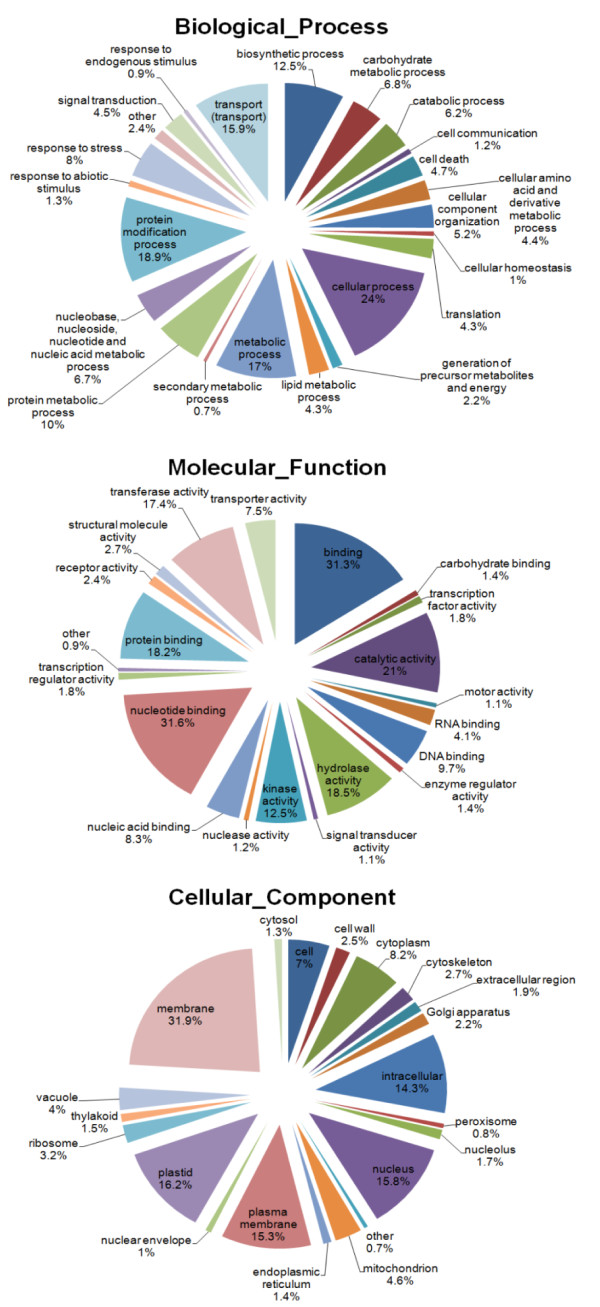
**GO annotation results of *O. longistaminata *pyrosequencing ESTs**. The total numbers of *O. longistaminata *unique EST sequences annotated for each category are 16036 for Biological Process, 20935 for Molecular Function and 13492 for Cellular Component.

The unique ESTs were further classified into Molecular Function, Biological Process and Cellular Components, according to the standard Gene Ontology terms (GO; http://www.geneontology.org). Only to 25,448 *O. longistaminata *sequence tags GO numbers were assigned, however a broad range of GO categories was covered: the percentage distribution of GO terms is shown in Figure 3. A total of 20,935 sequences could be assigned to the Molecular Function. Among them, nucleotide binding (31.6%) and binding (general, including small molecules) (31.3%) and catalytic activity (21%) were the most dominant categories. With regard to the category of Biological Process containing 16,036 ESTs, cellular processes (24%) were the most highly represented category. The following categories were protein modification processes (18.9%), metabolic processes (17%), transport (15.9%) and biosynthetic processes (12.5%). Under the category of Cellular Component, 31.9% of 13,492 ESTs were predicted as membrane proteins (general category including different organelles), followed by plastid (16.2%), nucleus (15.8%) and plasma membrane (15.3%) proteins.

### Biological Validation of Novel ESTs

Approximately 15.4% of the unique EST sequences detected in the *O. longistaminata *root transcriptome currently are not similar to rice sequences in databases. These may represent novel genes of *O. longistaminata *not present in *O. sativa*, or it may be possible that there are gaps existing in cultivated rice genome sequences, or a small portion of the unmapped sequences might have resulted from contamination by non-rice sources. A total of 14 novel ESTs were randomly selected for RT-PCR to determine the portion of potential novel genes originating from *O. longistaminata *rather other organisms in our transcript collection. RT-PCR experiments were conducted on RNAs derived from root tissue of clonally propagated *O. longistaminata *plants grown in soil in the phytotron. Of the 13 primer pairs for PCR, 10 generated RT-PCR products that were of the expected size and whose sequences were confirmed by Sanger sequencing. The results demonstrated that these 10 novel transcripts detected among the 454-ESTs are indeed expressed in *O. longistaminata *roots grown in soil (Figure 2A). Among another set of primer pairs for 19 additional ESTs, six yielded a positive result (Additional file 5). However, as conditions for PCR amplification cannot be optimized due to lack of intron-free template, these results may be an underestimation. To test for distribution of the putatively expressed genes among different accessions of the same species, *O. longistaminata *grains collected at the Okavango region of Namibia were used for gnotobiotic cultivation of seedlings in the phytotron, and pooled for analysis. From root RNA extracts, 5 of the 10 primer pairs yielded RT-PCR products with correct size whose sequences were validated by Sanger sequencing again. This confirmed that these fragments indeed originated from this species and not *e.g*. from root endophytes, and that their expression was conserved within the species. To control occurrence in the genome, Southern blot analysis was carried out from genomic DNA extracted from leaves of *O. longistaminata *accession IRGC 110404, and from *O. sativa*. Probes generated from two out of the 10 ESTs detected hybridizing fragments for wild but not for cultivated rice (Figure 2B). The results indicated that these 2 ESTs are indeed *O. longistaminata*-specific sequences. Based on these results, we estimated that a large subset of novel sequences was derived from *O. longistaminata*. The remaining novel EST sequences might be due to the contamination from other sources or due to the 454 sequencing artefacts.

## Conclusions

In this study, we present a large-scale EST dataset comprising 71,367 unique EST sequences derived from wild rice *O. longistaminata *by massively parallel pyrosequencing. Among them, 34,510 ESTs matched to known gene models, and 25,448 ESTs were annotated with GO terms. The comparative analysis between wild rice and two domesticated rice subspecies indicated that *O. longistaminata *had parallel similarity to *japonica *as to *indica *rice. Notably, a large amount of ESTs derived from *O. longistaminata *roots have not yet been detected as expressed in *O. sativa*, or did not show similarity to publicly available rice sequences or any other genes. Our data contribute to future annotation approaches of the *O. longistaminata *genome, to identification of *O. longistaminata*-specific genes and to the comparative study of the evolution among *Oryza *genus. These novel ESTs will particularly provide a basis for further identification of genes of *O. longistaminata *underlying adaptation to nutrient-limiting conditions. All EST obtained in this study is attached in the supplemental data (Additional file 6).

## Methods

### Plant materials

The *O. longistaminata *accession IRGC 110404 (short name Xa21) was grown under nitrogen-limiting conditions in soil without nitrogen fertilizer in the phytotron in Bremen. The soil (from Camargue) had a low percentage of total nitrogen (0,229%) and a high C/N ratio (25.5). The roots and leaves were harvested by snap-freezing in liquid nitrogen and prepared for RNA and DNA isolation, respectively. The seeds of *O. longistaminata *collected from Namibia were surface-sterilized [28] and cultured gnotobiotically in plant medium [29] supplemented with agar (4 g per L).

### RNA and DNA extraction, cDNA synthesis

The RNA was extracted by the CTAB method described by Chang et al. [30] from soil-grown roots and then purified using plant RNeasy columns (Qiagen, Hilden). The RNA from cultured seeds was isolated using TRIzol (Invitrogen) according to manufacturer's instructions. The quality of RNA was evaluated by a Bioanalyzer 2100 (Agilent Technologies, Santa Clara, CA). Genomic DNA was isolated by the CTAB method described by Allen et al. [31] from leaves. The concentration of DNA was determined spectrophotometrically and the quality of DNA was checked by agarose gel electrophoresis. cDNA was synthesized using the SMART PCR cDNA synthesis Kit (Clontech, Mountain View, CA). cDNA was purified by QIAquick spin columns (Qiagen, Hilden).

### 454 pyrosequencing, assembly and annotation

Synthesis of cDNA and normalization for pyrosequencing was carried out by MWG (Ebersberg, Germany) using RNA from roots of soil-grown plants without N-fertilizer. High quality polyA+ RNA was isolated from total RNA as template for first- and second-strand synthesis. By using a semirandom priming approach for both strands, an even shotgun-like distribution of cDNA fragments was achieved. The fragments were size-fractionated and normalised by denaturing and re-association. Approximately 10 μg of cDNAs were sheared by nebulisation and sequenced on a 454 GS-FLX pyrosequencing platform. A total of 337,830 raw reads were obtained. SeqClean software http://compbio.dfci.harvard.edu/tgi/software/ was applied to eliminate low quality sequences, poly A/T sequences, adaptor sequences. The cleaned sequences were subjected to the CAP3 program [32] for clustering and assembly with default parameters. All the consensus sequences were compared with NR database (GenBank). GO accessions were obtained via assignment of *Arabidopsis *gene identifiers with the strongest BLASTx alignments to the corresponding *O. longistaminata *ESTs. Comparison of the distribution of cellular component, biological processes or molecular function obtained using GO annotation was done using the GOSlim program http://www.geneontology.org.

The sequences are available at http://www.gabipd.org/ under the accession Xa21_454, and at GenBank (dbEST acc. No. HS317469 - HS388835).

### RT-PCR and Southern blot analyses

To validate the presence of novel ESTs detected by pyrosequencing in *O. longistaminata*, randomly selected sequences were used for expression analysis by RT-PCR (root RNA) and Southern blot (leaf DNA) analyses. About 100 ng total RNA was use to synthesize the first-strand cDNA by SuperScript™ II Reverse Transcriptase (Invitrogen, Carlsbad, CA) with Oligo(dT)12-18 primers. Specific primer pairs for cDNA amplification were designed by Primer3 software [33] according to the EST sequences. PCR was performed in a 50 μL reaction volume containing 1 μL cDNA, 1× PCR buffer [10 mM Tris-Hcl (pH 8.0), 1.5 mM MgCl2], 0.2 mM dNTPs, 0.2 μM of each primer, and 1.5 U Taq polymerase (MolTaq). The annealing temperature was 60°C for all primer pairs. After 5 min at 94°C, 35 cycles were carried out with 45 s at 94°C, 45 s at 60°C, 1 min at 72°C for extension and final step of 10 min at 72°C. The PCR products were purified and sequenced by the Sanger method (LGC Genomics, Germany). For Southern blot analysis, 5 μg of genomic DNA was used for restriction endonuclease digestion with HindIII and subjected to Southern blot analysis with digoxygenin-labeled probes according to the protocol described by Neuhaus-Url et al. [34].

## Authors' contributions

HY carried out *O. longistaminata *root RNA isolation, RT-PCR and participated in sequence analyses, and drafted the manuscript; LH carried out Southern analysis and participated in sequence analyses; TH prepared the plant materials used in this study and co-designed the experiment; BR designed the experiment and assisted in the manuscript preparation. All authors read and approved the final manuscript.

## Supplementary Material

Additional file 1 Source of *O. longistaminata *root ESTs. Click here for file

Additional file 2 **Characterization of O. longistaminata ESTs before and after assembly**. Click here for file

Additional file 3 **Summary of component reads per assembly**. Click here for file

Additional file 4 **Consensus sequences specifically matched to indica or japonica genome sequences**. Click here for file

Additional file 5 *O. longistaminata *ESTs analyzed by RT-PCR from cDNA of pot-grown *O. longistaminata *rice roots. Click here for file

Additional file 6 **Assembled pyrosequencing ESTs**. The data represent all the assembled pyrosequencing ESTs. Click here for file
