# Supplementary material for: Global characterization of the root transcriptome of a wild species of rice, Oryza longistaminata, by deep sequencing
Source: BMC Genomics. 2010 Dec 15;11:705. doi: 10.1186/1471-2164-11-705 (PMC3016420; doi:10.1186/1471-2164-11-705)
Supplement: Additional file 1 — Source of O. longistaminata root ESTs. [file 1471-2164-11-705-S1.PDF]

Additional file 1: Source of *O. longistaminata* root ESTs

A **B** **C**

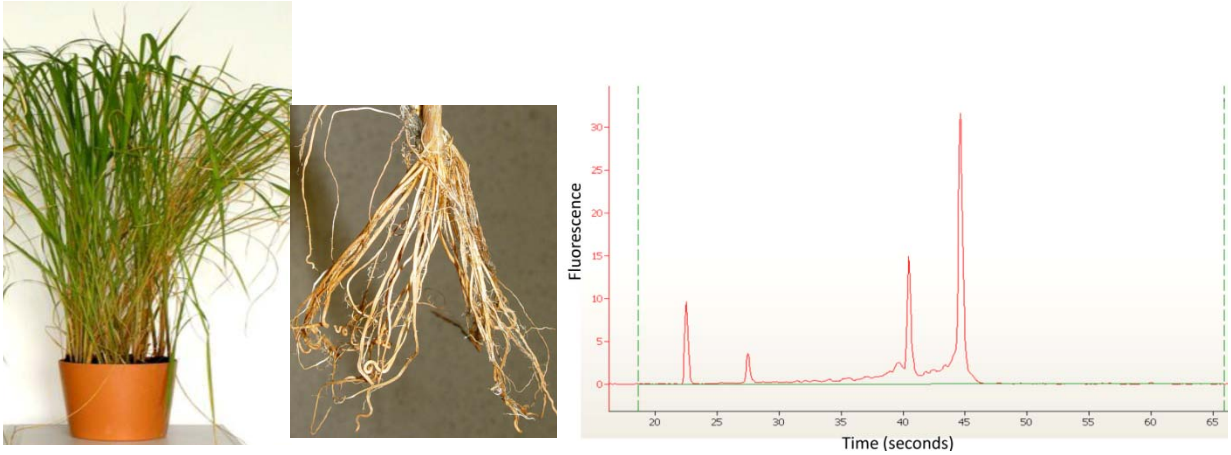

(A) The *O. longistaminata* accession IRGC 110404 was grown under nitrogen-limiting conditions in pots.

(B) Root system of *O. longistaminata* used for RNA extraction

(C) Total RNA was extracted by the CTAB method from wild rice root tissue.

The quality of RNA was evaluated using the Agilent Bioanalyzer 2100.
