# Supplementary material for: Global characterization of the root transcriptome of a wild species of rice, Oryza longistaminata, by deep sequencing
Source: BMC Genomics. 2010 Dec 15;11:705. doi: 10.1186/1471-2164-11-705 (PMC3016420; doi:10.1186/1471-2164-11-705)
Supplement: Additional file 2 — Characterization of O. longistaminata ESTs before and after assembly. [file 1471-2164-11-705-S2.PDF]

Additional file 2:

Table S1: Characterization of *O. longistaminata* ESTs before and after assembly

Items	Total length (Mb)	Number of reads	Average length (bp)	Maximum length (bp)	Number of NR match
Raw sequences	87.3	337,830	2,589	925	
Cleaned sequences	66.7	337,471	197	393	
Singlets	6.5	30,178	215	393	11,215
Contigs	12.3	264,373	299	2082	23,295
Unique ESTs	18.8	294,551	263	2082	34,510
