# Supplementary material for: Global characterization of the root transcriptome of a wild species of rice, Oryza longistaminata, by deep sequencing
Source: BMC Genomics. 2010 Dec 15;11:705. doi: 10.1186/1471-2164-11-705 (PMC3016420; doi:10.1186/1471-2164-11-705)
Supplement: Additional file 3 — Summary of component reads per assembly. [file 1471-2164-11-705-S3.PDF]

Additional file 3:

Table S2: Summary of component reads per assembly

**Number of reads**	**Number of contigs**
2	11,949 (29.0%)
3	7,226 (17.5%)
4	4,766 (11.6%)
5	3,274 (2.90%)
6	2,464 (6.0%)
7	1,903 (4.6%)
8	1,449 (3.5%)
9	1,149 (2.8%)
10	915 (2.2%)
11-20	4,155 (10.1%)
21-30	1,073 (2.6%)
31-40	495 (1.2%)
41-50	195 (0.5%)
51-297	176 (0.4%)
