# Supplementary material for: Global characterization of the root transcriptome of a wild species of rice, Oryza longistaminata, by deep sequencing
Source: BMC Genomics. 2010 Dec 15;11:705. doi: 10.1186/1471-2164-11-705 (PMC3016420; doi:10.1186/1471-2164-11-705)
Supplement: Additional file 4 — Consensus sequences specifically matched to indica or japonica genome sequences. [file 1471-2164-11-705-S4.PDF]

Additional file 4 | **Consensus sequences matching to indica or japonica genome sequences**

Contig/singleton	japonica-hit	Identity	E-value	Score	indica-hit	Identity	E-value	Score
Xa21_261					Chr02	98.32	0	998
Xa21_423	chr12	89.26	8.00E-41	169				
Xa21_616	chr01	100	4.00E-130	466				
Xa21_637					Chr10	86.96	4.00E-07	58.1
Xa21_801					Chrunknown	93.24	2.00E-86	321
Xa21_1045					Chrunknown	99.46	3.00E-98	361
Xa21_1357	chr01	99.35	1.00E-167	591				
Xa21_1509	chr11	94.71	6.00E-70	266				
Xa21_1574					Chr06	98.1	0	1257
Xa21_1666					Chr07	93.71	0	694
Xa21_1864	chr03	88.8	3.00E-110	400				
Xa21_1871	chr08	99.55	7.00E-121	436				
Xa21_1920					Chr01	86.59	3.00E-12	75.8
Xa21_2040					Chr02	90.34	3.00E-60	234
Xa21_2052	chr06	99.31	0	833				
Xa21_2193					Chr05	98.38	9.00E-123	442
Xa21_2413					Chr04	99.24	3.00E-63	244
Xa21_2876					Chr02	97.88	0	722
Xa21_2906					Chr07	100	5.00E-153	542
Xa21_3370					Chr04	98.6	5.00E-150	533
Xa21_3385					Chr02	82.98	1.00E-07	60
Xa21_3401	chr01	100	7.00E-42	172				
Xa21_3737					Chr11	92.5	3.00E-06	56.1
Xa21_3864					Chr09	99.29	0	773
Xa21_3888					Chrunknown	97.84	6.00E-114	412
Xa21_4280					Chrunknown	87.74	4.00E-22	107
Xa21_4457	chr06	99.75	0	787				
Xa21_4579	chr12	97.85	0	666				
Xa21_4591	chr01	97.47	0	731				
Xa21_4595	chr12	96.37	2.00E-172	607				

Xa21_4666	chr04	93.96	7.00E-110	398				
Xa21_4752	chr11	85.93	9.00E-42	172				
Xa21_4764	chr02	95	3.00E-104	381				
Xa21_4773	chr12	98.14	3.00E-165	583				
Xa21_4927	chr08	98.94	0	674				
Xa21_5145	chr03	88.8	1.00E-27	125				
Xa21_5289					Chr09	89.66	4.00E-10	67.9
Xa21_5347	chr07	89.09	4.00E-06	54				
Xa21_5350	chr12	87.01	3.00E-08	61.9				
Xa21_5412					Chr12	99.36	4.00E-80	301
Xa21_5558					Chr04	99.15	1.00E-124	448
Xa21_5589					Chrunknown	99.36	8.00E-174	612
Xa21_5849	chr01	86.67	1.00E-06	56				
Xa21_6038					Chr09	99.43	0	1316
Xa21_6085	chr01	98.88	2.00E-91	337				
Xa21_6108	chr11	98.12	0	870				
Xa21_6112					Chr12	91.11	5.00E-07	58
Xa21_6164					Chrunknown	100	1.00E-113	412
Xa21_6298					Chr03	98.31	1.00E-86	321
Xa21_6399					Chr04	100	0	658
Xa21_6451	chr05	99.41	4.00E-88	327				
Xa21_6502					Chr11	99.16	3.00E-58	228
Xa21_6638	chr02	99.48	0	747				
Xa21_6724					Chr05	93.75	7.00E-16	87.9
Xa21_6786	chr12	90.61	3.00E-57	224				
Xa21_6805	chr07	98.94	0	718				
Xa21_6903					Chr04	98.64	3.00E-156	553
Xa21_7122	chr06	100	0	686				
Xa21_7250					Chr05	95.19	1.00E-37	159
Xa21_7386	chr06	96.57	3.00E-165	583				
Xa21_7464					Chrunknown	99.47	0	731
Xa21_7561					Chr02	98.16	1.00E-109	398
Xa21_7603	chr12	98.92	0	697				

Xa21_7758					Chr06	98.67	1.00E-146	521
Xa21_7907	chr08	99.6	1.00E-136	488				
Xa21_7936					Chr12	93.53	9.00E-76	286
Xa21_8151					Chr06	90.32	6.00E-116	420
Xa21_8163					Chr03	89.68	5.00E-34	147
Xa21_8168	chr02	83.83	2.00E-22	109				
Xa21_8284	chr03	97.87	1.00E-66	256				
Xa21_8483					Chr11	100	8.00E-132	472
Xa21_8535	chr08	99.34	9.00E-153	541				
Xa21_8574					Chrunknown	100	2.00E-36	155
Xa21_8605					Chrunknown	98.32	3.00E-154	547
Xa21_8661	chr03	98.02	0	826				
Xa21_8666					Chr06	88.66	2.00E-80	301
Xa21_8910	chr04	96.75	2.00E-51	204				
Xa21_8978	chr11	99.79	0	955				
Xa21_9018	chr03	99.43	2.00E-91	337				
Xa21_9192	chr02	99.69	0	641				
Xa21_9227					Chrunknown	99.74	0	743
Xa21_9230					Chr07	88.5	4.00E-54	214
Xa21_9357					Chr02	96.92	1.00E-108	394
Xa21_9488					Chr04	82.33	6.00E-20	101
Xa21_9517	chr09	94.09	7.00E-176	618				
Xa21_9590	chr01	98.94	1.00E-151	537				
Xa21_9671	chr12	86.59	2.00E-56	221				
Xa21_9764	chr06	95	1.00E-102	375				
Xa21_9804					Chr10	98.26	1.00E-121	438
Xa21_9842	chr07	98.92	2.00E-150	534				
Xa21_9896					Chr09	100	6.00E-164	579
Xa21_9954					Chr03	84.23	1.00E-38	163
Xa21_9980					Chrunknown	100	1.00E-90	335
Xa21_10088	chr11	100	1.00E-126	454				
Xa21_10219	chr08	94.47	1.00E-102	375				
Xa21_10331					Chr06	100	6.00E-74	280

Xa21_10347					Chrunknown	93.49	4.00E-86	320
Xa21_10381					Chr04	99.58	5.00E-130	466
Xa21_10657	chr08	97.37	7.00E-51	202				
Xa21_10672					Chr05	98.98	4.00E-157	557
Xa21_10722	chr10	99.09	7.00E-116	418				
Xa21_10768					Chr01	99.08	2.00E-52	208
Xa21_10815	chr04	98.82	1.00E-86	321				
Xa21_10823	chr12	92.2	2.00E-70	268				
Xa21_10835	chr12	94.19	5.00E-27	123				
Xa21_10944	chr01	98.19	1.00E-79	297				
Xa21_11024					Chr08	100	2.00E-107	391
Xa21_11051					Chr07	94.61	2.00E-85	317
Xa21_11179					Chr04	99.33	0	858
Xa21_11474	chr08	94.64	4.00E-40	167				
Xa21_11535	chr12	97.11	0	652				
Xa21_11544					Chrunknown	98.8	7.00E-130	466
Xa21_11596	chr07	100	3.00E-149	529				
Xa21_11600	chr04	98.63	9.00E-150	531				
Xa21_11636					Chrunknown	96.8	2.00E-52	208
Xa21_11837					Chr12	99.15	1.00E-124	448
Xa21_11883	chr12	97.67	5.00E-104	379				
Xa21_11934	chr06	93.9	7.00E-64	246				
Xa21_12002	chr10	98.3	1.00E-87	325				
Xa21_12034					Chr02	98.33	6.00E-90	333
Xa21_12174					Chrunknown	99.58	2.00E-129	464
Xa21_12187					Chr04	100	1.00E-74	281
Xa21_12195	chr01	84.16	5.00E-12	73.8				
Xa21_12196	chr08	99.72	0	682				
Xa21_12198					Chr04	98.64	5.00E-114	412
Xa21_12401	chr04	100	0	759				
Xa21_12511					Chr06	99.17	2.00E-129	464
Xa21_12759					Chr02	97.69	3.00E-60	234
Xa21_12891	chr04	97.32	5.00E-50	198				

Xa21_13266	chr01	99.05	6.00E-111	402				
Xa21_13367					Chr12	100	5.00E-91	337
Xa21_13535					Chr09	99.43	3.00E-79	297
Xa21_13638					Chr04	97.66	1.00E-115	418
Xa21_13639					Chrunknown	98.9	0	676
Xa21_13685					Chrunknown	98.55	3.00E-106	387
Xa21_13793	chr08	100	6.00E-52	206				
Xa21_13900					Chr04	97.75	0	844
Xa21_13928					Chr10	100	6.00E-55	216
Xa21_13954	chr04	98.8	5.00E-37	157				
Xa21_14208					Chrunknown	94.66	6.00E-90	333
Xa21_14222	chr01	94.59	1.00E-85	319				
Xa21_14573	chr03	99.62	3.00E-146	519				
Xa21_14574					Chrunknown	84.85	4.00E-16	87.7
Xa21_14661	chr01	97.67	1.00E-80	301				
Xa21_14717					Chr03	80.68	9.00E-14	79.8
Xa21_14864					Chrunknown	96.01	3.00E-128	460
Xa21_14898					Chrunknown	93.59	4.00E-22	107
Xa21_14899	chr08	97.86	1.00E-142	507				
Xa21_14941	chr01	100	1.00E-126	454				
Xa21_14984	chr06	99.82	0	1078				
Xa21_15147					Chr04	99.21	0	731
Xa21_15239					Chr10	98.65	4.00E-115	416
Xa21_15247					Chr04	98.77	1.00E-127	458
Xa21_15260	chr06	98.4	0	921				
Xa21_15280	chr02	99.16	9.00E-128	458				
Xa21_15358	chr07	99.07	4.00E-99	363				
Xa21_15403					Chr04	99.11	0	649
Xa21_15406	chr01	98.9	0	695				
Xa21_15447					Chr03	95.15	3.00E-94	347
Xa21_15453	chr11	98.96	3.00E-100	366				
Xa21_15564					Chrunknown	95.37	2.00E-42	174
Xa21_15624					Chrunknown	98.05	2.00E-102	375

Xa21_15648	chr05	91.92	9.00E-97	355				
Xa21_15961					Chrunknown	97.64	0	846
Xa21_16040					Chrunknown	99.57	2.00E-126	454
Xa21_16124					Chr05	91.84	2.00E-07	58
Xa21_16129					Chr12	99.14	0	660
Xa21_16210	chr08	98.24	2.00E-113	410				
Xa21_16448					Chrunknown	100	2.00E-151	537
Xa21_16492					Chrunknown	100	4.00E-165	583
Xa21_16507					Chrunknown	100	5.00E-143	509
Xa21_16878					Chr04	98.72	2.00E-166	587
Xa21_16940					Chrunknown	98.2	5.00E-49	197
Xa21_17086					Chr07	96.85	1.00E-105	385
Xa21_17201	chr06	92.96	1.00E-80	301				
Xa21_17374	chr07	99.15	1.00E-123	444				
Xa21_17602					Chr07	83.48	3.00E-13	77.8
Xa21_17723					Chrunknown	96.95	4.00E-93	343
Xa21_17843	chr11	98.93	6.00E-152	538				
Xa21_17849					Chrunknown	100	2.00E-64	248
Xa21_17857	chr02	97.58	1.00E-101	371				
Xa21_18010	chr05	94.54	0	656				
Xa21_18016	chr06	99.06	9.00E-112	404				
Xa21_18073	chr06	94.71	1.00E-67	258				
Xa21_18102	chr01	98.1	9.00E-106	385				
Xa21_18103					Chr12	97.56	4.00E-56	220
Xa21_18396					Chr12	97	4.00E-93	343
Xa21_18415					Chr12	99.47	4.00E-99	363
Xa21_18497	chr02	97.87	3.00E-41	170				
Xa21_18695					Chr11	84.31	4.00E-10	67.9
Xa21_18722					Chrunknown	98.69	1.00E-114	414
Xa21_18779	chr11	83.05	1.00E-12	75.8				
Xa21_18875					Chr12	99.24	4.00E-66	254
Xa21_18920	chr01	97.55	1.00E-77	291				
Xa21_18922					Chr01	99.47	3.00E-100	367

Xa21_19034					Chrunknown	96.88	1.00E-106	389
Xa21_19151					Chr02	97.35	2.00E-92	341
Xa21_19165					Chr06	91.45	1.00E-35	153
Xa21_19221	chr11	98.29	1.00E-74	281				
Xa21_19223					Chr02	98.74	1.00E-79	299
Xa21_19262	chr08	100	2.00E-122	440				
Xa21_19297	chr10	100	4.00E-58	226				
Xa21_19420	chr05	99.15	2.00E-55	218				
Xa21_19593					Chrunknown	92.73	1.00E-112	408
Xa21_19599					Chr11	92.09	2.00E-27	125
Xa21_19639	chr03	99.35	5.00E-79	295				
Xa21_19669	chr07	80.81	8.00E-14	79.8				
Xa21_19702					Chr05	88.24	6.00E-06	54
Xa21_19811	chr11	80.59	2.00E-12	75.8				
Xa21_19859	chr04	99.61	3.00E-137	490				
Xa21_20080					Chr03	99.58	1.00E-128	462
Xa21_20111					Chr11	92.42	5.00E-17	91.7
Xa21_20214	chr03	96.55	4.00E-35	149				
Xa21_20274	chr06	84.9	2.00E-45	184				
Xa21_20277	chr08	95.56	2.00E-133	477				
Xa21_20445	chr06	97.75	2.00E-38	161				
Xa21_20606					Chr04	98.56	4.00E-143	509
Xa21_20672	chr08	95.87	8.00E-94	345				
Xa21_20711	chr02	98.76	2.00E-81	303				
Xa21_20736	chr07	98.86	7.00E-142	505				
Xa21_21033					Chrunknown	98.93	0	1065
Xa21_21051	chr11	100	0	668				
Xa21_21070					Chr10	100	9.00E-119	428
Xa21_21176	chr01	97.74	5.00E-60	232				
Xa21_21304					Chrunknown	98.04	4.00E-74	280
Xa21_21323					Chr02	98.43	1.00E-119	432
Xa21_21382	chr05	93.7	5.00E-89	329				
Xa21_21485					Chr11	82.05	2.00E-09	65.9

Xa21_21501					Chrunknown	99.09	3.00E-175	617
Xa21_21518					Chrunknown	98.79	3.00E-161	570
Xa21_21727					Chr03	97.47	9.00E-30	133
Xa21_21742					Chrunknown	99.57	0	900
Xa21_21782					Chrunknown	97.52	6.00E-78	293
Xa21_21791	chr12	99.16	3.00E-124	446				
Xa21_21825	chr12	99.17	2.00E-128	460				
Xa21_22025	chr01	99.15	5.00E-126	452				
Xa21_22135	chr03	99.32	1.00E-74	281				
Xa21_22143					Chr04	99.3	1.00E-72	276
Xa21_22210	chr04	100	3.00E-90	333				
Xa21_22212					Chrunknown	91.13	1.00E-68	262
Xa21_22239					Chr02	99.01	1.00E-105	385
Xa21_22361					Chr10	93.99	4.00E-78	293
Xa21_22499					Chr01	91.8	6.00E-82	306
Xa21_22558					Chr08	92.15	6.00E-64	246
Xa21_22566	chr08	97.82	2.00E-163	577				
Xa21_22631					Chr04	99.13	2.00E-56	220
Xa21_22795	chr02	83.41	4.00E-34	147				
Xa21_23049					Chr05	98.27	2.00E-101	371
Xa21_23138	chr09	87.22	4.00E-28	127				
Xa21_23223					Chrunknown	100	4.00E-121	436
Xa21_23325	chr06	81.31	4.00E-06	54				
Xa21_23394	chr06	99.27	1.00E-148	527				
Xa21_23445					Chr06	91.62	8.00E-64	246
Xa21_23463					Chr01	99.55	6.00E-120	432
Xa21_23564	chr01	95.52	3.00E-53	210				
Xa21_23568	chr08	86.01	1.00E-52	208				
Xa21_23867					Chr02	98.17	1.00E-65	252
Xa21_23899	chr02	94.44	2.00E-48	194				
Xa21_24104					Chr01	99.07	5.00E-114	412
Xa21_24116					Chrunknown	99.54	4.00E-118	426
Xa21_24407	chr03	98.94	5.00E-98	359				

Xa21_24738	chr05	99.52	6.00E-113	408				
Xa21_24779	chr11	96.48	6.00E-104	379				
Xa21_24841					Chr02	98.24	4.00E-84	313
Xa21_24848	chr12	99.03	4.00E-108	392				
Xa21_24852	chr08	98.65	9.00E-153	541				
Xa21_24890	chr12	99.52	6.00E-113	408				
Xa21_24959					Chrunknown	92.96	3.00E-81	303
Xa21_25089					Chrunknown	98.25	2.00E-156	553
Xa21_25122	chr11	98.03	1.00E-101	371				
Xa21_25206	chr01	96.64	1.00E-51	204				
Xa21_25288					Chr12	97.25	3.00E-103	377
Xa21_25300					Chr10	98.97	3.00E-156	553
Xa21_25307	chr07	100	7.00E-141	502				
Xa21_25457	chr11	98.31	3.00E-109	396				
Xa21_25517					Chrunknown	95.98	1.00E-89	331
Xa21_25577	chr06	94.29	8.00E-06	54				
Xa21_25578					Chr03	91.36	5.00E-77	289
Xa21_25620					Chr10	99.28	3.00E-71	270
Xa21_25654					Chr09	99.07	1.00E-111	404
Xa21_25659					Chr03	98.68	1.00E-118	428
Xa21_25701					Chr10	100	5.00E-114	412
Xa21_25803					Chr04	90.79	2.00E-42	174
Xa21_25858					Chr08	96.46	1.00E-105	385
Xa21_25865					Chrunknown	100	8.00E-107	389
Xa21_25929	chr06	99.61	3.00E-140	500				
Xa21_26099					Chrunknown	94.29	8.00E-06	54
Xa21_26206					Chr03	89.41	4.00E-19	97.6
Xa21_26424					Chrunknown	90.94	4.00E-91	337
Xa21_26609	chr12	99.55	3.00E-121	436				
Xa21_26796					Chr06	98.33	4.00E-143	509
Xa21_26844					Chrunknown	99.15	0	678
Xa21_26904	chr02	99.47	5.00E-101	369				
Xa21_26982					Chr08	99.07	7.00E-170	599

Xa21_27266					Chr12	100	4.00E-99	363
Xa21_27412					Chr10	99.55	6.00E-120	432
Xa21_27440					Chr03	98.68	8.00E-33	143
Xa21_27463	chr10	92.47	1.00E-67	258				
Xa21_27545					Chrunknown	100	9.00E-76	285
Xa21_27698	chr03	91.53	7.00E-84	312				
Xa21_27793					Chr11	100	7.00E-110	398
Xa21_27815					Chr11	98.91	4.00E-94	347
Xa21_27898	chr10	96.81	1.00E-130	468				
Xa21_27937					Chr03	100	8.00E-57	222
Xa21_28056	chr12	98.81	0	1267				
Xa21_28127	chr11	100	3.00E-51	202				
Xa21_28222	chr02	89.8	3.00E-07	58				
Xa21_28237					Chr11	85.71	4.00E-06	54
Xa21_28304	chr07	98.95	1.00E-98	361				
Xa21_28385	chr11	98.35	2.00E-57	224				
Xa21_28393	chr06	98.16	8.00E-166	585				
Xa21_28408	chr01	89.04	2.00E-12	73.8				
Xa21_28461	chr01	98.93	7.00E-148	525				
Xa21_28553					Chrunknown	97.44	8.00E-32	139
Xa21_28753					Chr11	97.69	1.00E-168	595
Xa21_28772	chr06	100	1.00E-20	101				
Xa21_29070	chr08	98.4	2.00E-57	224				
Xa21_29262					Chr10	99.13	1.00E-121	438
Xa21_29266					Chr04	100	9.00E-111	402
Xa21_29279					Chr02	99.15	1.00E-57	224
Xa21_29364	chr03	92.68	6.00E-23	109				
Xa21_29565	chr08	98.58	2.00E-109	396				
Xa21_29587	chr12	92	3.00E-10	67.9				
Xa21_29620					Chrunknown	98	1.00E-69	266
Xa21_29788					Chr10	99.37	9.00E-82	305
Xa21_29912	chr12	95.69	2.00E-116	420				
Xa21_30135					Chr09	97.2	7.00E-120	432

Xa21_30145					Chrunknown	100	2.00E-24	115
Xa21_30146					Chr10	98.21	6.00E-111	402
Xa21_30370					Chrunknown	92.22	5.00E-22	107
Xa21_30418	chr06	97.17	7.00E-107	389				
Xa21_30516					Chr02	99.59	3.00E-134	480
Xa21_30603	chr07	99.6	9.00E-123	441				
Xa21_30663	chr01	97.97	3.00E-93	343				
Xa21_30674					Chr01	93.33	1.00E-81	305
Xa21_30727					Chrunknown	100	3.00E-64	248
Xa21_30734					Chrunknown	99.62	1.00E-143	511
Xa21_30875	chr06	95.42	1.00E-64	248				
Xa21_31130					Chrunknown	95.89	8.00E-61	236
Xa21_31136	chr01	100	5.00E-58	226				
Xa21_31271	chr03	90.91	9.00E-07	56				
Xa21_31306					Chrunknown	84.55	2.00E-15	85.7
Xa21_31339	chr05	98.76	5.00E-173	609				
Xa21_31362					Chrunknown	88.71	5.00E-28	127
Xa21_31521					Chr03	96.76	8.00E-101	369
Xa21_31542	chr11	92.8	7.00E-42	172				
Xa21_31544					Chr05	88.03	3.00E-35	151
Xa21_31692					Chr01	93.06	3.00E-82	307
Xa21_31802					Chr12	99.12	2.00E-120	434
Xa21_32072					Chr06	100	4.00E-90	333
Xa21_32125	chr06	100	1.00E-123	444				
Xa21_32134	chr12	91.83	1.00E-71	272				
Xa21_32246	chr11	98.17	3.00E-50	200				
Xa21_32280	chr06	88.14	4.00E-06	54				
Xa21_32322	chr01	100	9.00E-60	232				
Xa21_32405	chr11	100	8.00E-125	448				
Xa21_32595	chr06	100	4.00E-46	186				
Xa21_32644					Chr05	97.47	5.00E-68	260
Xa21_32712					Chr03	100	2.00E-123	444
Xa21_32897	chr06	100	5.00E-123	442				

Xa21_32913	chr02	97.65	5.00E-95	349				
Xa21_32938	chr06	99.31	2.00E-73	278				
Xa21_32941					Chrunknown	85.06	1.00E-31	139
Xa21_32975	chr03	93.28	8.00E-88	325				
Xa21_33000	chr10	98.26	3.00E-115	416				
Xa21_33011					Chr03	97.41	1.00E-90	335
Xa21_33065					Chr10	99.55	6.00E-120	432
Xa21_33217	chr04	100	2.00E-122	440				
Xa21_33339					Chr02	100	3.00E-112	406
Xa21_33480					Chr11	100	9.00E-42	172
Xa21_33592					Chr01	97.71	5.00E-108	392
Xa21_33648					Chr10	100	7.00E-126	452
Xa21_33760	chr02	100	2.00E-122	440				
Xa21_33834					Chr03	94.44	6.00E-46	186
Xa21_33932	chr09	88.42	3.00E-47	190				
Xa21_34051					Chrunknown	96.07	7.00E-145	515
Xa21_34062	chr12	100	3.00E-44	180				
Xa21_34076					Chrunknown	94.91	2.00E-92	341
Xa21_34202					Chr04	95.39	3.00E-92	340
Xa21_34240					Chrunknown	92.02	2.00E-76	287
Xa21_34261	chr07	100	3.00E-118	426				
Xa21_34265					Chr02	99.53	5.00E-114	412
Xa21_34297					Chr04	94.44	6.00E-89	329
Xa21_34351	chr01	98.65	2.00E-113	410				
Xa21_34365					Chr03	100	1.00E-53	212
Xa21_34397					Chrunknown	99.53	3.00E-115	416
Xa21_34543					Chr02	98.6	5.00E-108	392
Xa21_34577					Chr01	100	5.00E-117	422
Xa21_34583	chr08	99.07	1.00E-111	404				
Xa21_34615	chr01	99.07	2.00E-113	410				
Xa21_34877	chr07	99.53	3.00E-116	420				
Xa21_34925					Chr05	100	1.00E-56	222
Xa21_35121	chr12	100	1.00E-118	428				

Xa21_35198					Chrunknown	88.18	3.00E-23	111
Xa21_35258					Chrunknown	88.24	2.00E-64	248
Xa21_35332					Chrunknown	93.65	3.00E-44	180
Xa21_35433	chr10	98.15	6.00E-107	389				
Xa21_35496	chr06	95.26	6.00E-82	305				
Xa21_35523	chr11	100	8.00E-70	265				
Xa21_35549	chr11	95.02	1.00E-95	351				
Xa21_35670					Chrunknown	84.4	2.00E-14	81.8
Xa21_35683					Chrunknown	95.75	4.00E-90	333
Xa21_35748					Chrunknown	95.92	2.00E-14	81.8
Xa21_35819					Chr11	98.08	4.00E-102	373
Xa21_35845	chr02	96.65	2.00E-113	410				
Xa21_35882					Chr01	99.02	7.00E-107	389
Xa21_35933	chr04	98.92	3.00E-131	470				
Xa21_36009	chr04	97.71	7.00E-59	229				
Xa21_36112					Chrunknown	98.45	4.00E-93	343
Xa21_36560	chr07	88.41	4.00E-12	73.8				
Xa21_36597					Chrunknown	92.68	4.00E-07	58.1
Xa21_36830	chr09	94.44	4.00E-40	167				
Xa21_36848	chr09	99.19	5.00E-61	236				
Xa21_36895	chr05	96.12	9.00E-54	212				
Xa21_37013					Chr04	97.64	1.00E-107	391
Xa21_37094	chr05	98.29	3.00E-118	426				
Xa21_37097					Chr11	97.18	2.00E-127	457
Xa21_37101					Chr11	100	4.00E-84	313
Xa21_37110					Chrunknown	97.78	1.00E-102	375
Xa21_37140					Chr11	97.87	3.00E-90	333
Xa21_37351					Chr04	100	7.00E-151	535
Xa21_37369	chr01	92.98	1.00E-14	81.8				
Xa21_37783	chr04	96	2.00E-85	317				
Xa21_38033	chr08	97.22	4.00E-100	366				
Xa21_38108					Chrunknown	97.8	2.00E-38	161
Xa21_38133					Chr02	98.69	2.00E-119	430

Xa21_38235					Chr01	99.63	1.00E-146	521
Xa21_38268	chr01	97.2	2.00E-172	607				
Xa21_38339					Chr06	96.97	6.00E-74	280
Xa21_38363	chr08	99.07	4.00E-113	409				
Xa21_38415	chr10	96.86	6.00E-104	379				
Xa21_38599					Chr01	98.8	3.00E-84	313
Xa21_38689					Chr04	97.85	3.00E-42	174
Xa21_38776					Chr04	94.55	6.00E-114	412
Xa21_39075					Chrunknown	84.78	2.00E-11	71.9
Xa21_39128					Chr07	92.58	4.00E-78	293
Xa21_39192	chr04	97.37	2.00E-30	135				
Xa21_39223	chr12	100	2.00E-71	270				
Xa21_39230	chr09	90.91	2.00E-44	180				
Xa21_39277					Chrunknown	97.78	2.00E-63	244
Xa21_39286	chr12	85.29	1.00E-06	56				
Xa21_39313	chr08	93.46	9.00E-82	305				
Xa21_39488	chr11	96.99	0	646				
Xa21_39542					Chr02	98.6	9.00E-111	402
Xa21_39545	chr02	96.53	2.00E-69	264				
Xa21_39699					Chrunknown	99.02	7.00E-46	187
Xa21_39708					Chrunknown	95.45	2.00E-54	214
Xa21_39712					Chrunknown	88.54	3.00E-19	97.6
Xa21_39751					Chrunknown	88.89	3.00E-11	71.9
Xa21_39755	chr11	96.88	1.00E-06	56				
Xa21_39819					Chrunknown	88.89	2.00E-09	65.9
Xa21_39855					Chrunknown	97.48	2.00E-138	494
Xa21_39944					Chrunknown	87.8	5.00E-35	151
Xa21_40023					Chr02	98.37	7.00E-120	432
Xa21_40056					Chrunknown	100	1.00E-84	315
Xa21_40146	chr06	87.5	2.00E-42	174				
Xa21_40184					Chr06	91.38	1.00E-33	145
Xa21_40215					Chr04	97.14	2.00E-45	184
Xa21_40370	chr01	100	2.00E-47	190				

Xa21_40474	chr01	99.35	2.00E-79	297				
Xa21_40482	chr01	98.97	9.00E-46	184				
Xa21_40497					Chrunknown	92.62	2.00E-41	170
Xa21_40574					Chrunknown	98.04	4.00E-97	357
Xa21_40642	chr03	99.05	7.00E-51	202				
Xa21_40764					Chrunknown	91.15	1.00E-33	145
Xa21_40840					Chr03	97.79	2.00E-115	418
Xa21_40883	chr08	97.85	5.00E-41	168				
Xa21_41007	chr01	97.74	5.00E-60	232				
Xa21_41303	chr08	98.48	3.00E-65	250				
Xa21_41428	chr12	98.2	1.00E-85	317				
Xa21_41445					Chr03	89.95	8.00E-58	226
Xa21_41631					Chr01	100	1.00E-38	161
Xa21_41663	chr11	97.14	1.00E-38	161				
Xa21_41817					Chr03	97.4	6.00E-31	137
Xa21_41869	chr06	98.72	1.00E-148	527				
Xa21_42035	chr03	100	2.00E-32	141				
Xa21_42297	chr12	97.93	6.00E-120	432				
Xa21_42327					Chr03	99.44	4.00E-94	347
Xa21_42369	chr09	98.65	0	688				
Xa21_42436	chr11	98.37	1.00E-170	601				
Xa21_42583	chr07	97.52	3.00E-115	416				
Xa21_42838	chr09	95.06	6.00E-29	129				
Xa21_43099					Chr01	98.7	9.00E-116	418
Xa21_43284					Chrunknown	98.86	1.00E-137	492
Xa21_43423					Chr03	93.06	2.00E-16	88.1
Xa21_43534					Chr04	99.6	1.00E-136	488
Xa21_43823	chr06	99.18	2.00E-60	234				
Xa21_43923	chr06	94.48	9.00E-56	218				
Xa21_43937					Chr02	97.91	4.00E-118	426
Xa21_44012					Chr03	97.22	4.00E-45	184
Xa21_44110					Chrunknown	99.42	2.00E-88	327
Xa21_44333	chr09	95.73	6.00E-101	369				

Xa21_44334	chr09	85.06	5.00E-24	113				
Xa21_44336					Chr09	99.12	2.00E-120	434
Xa21_44368	chr03	99.1	3.00E-118	426				
Xa21_44479	chr09	87.42	9.00E-35	149				
Xa21_44647					Chr02	97.3	2.00E-51	204
Xa21_44651					Chr05	98.39	2.00E-54	214
Xa21_44662	chr11	91.3	7.00E-19	95.6				
Xa21_44753	chr03	98.45	3.00E-96	353				
Xa21_44925	chr06	91.3	2.00E-08	60.4				
Xa21_44969	chr07	98.59	7.00E-28	125				
Xa21_45055	chr08	100	2.00E-13	77.8				
Xa21_50174					Chrunknown	86.19	3.00E-38	161
Xa21_50191	chr07	97.47	3.00E-30	133				
Xa21_50296					Chrunknown	95.04	3.00E-103	377
Xa21_50346					Chr12	100	6.00E-120	432
Xa21_50372	chr08	92.47	2.00E-24	115				
Xa21_50399					Chr09	99.55	4.00E-121	436
Xa21_50403					Chrunknown	92.39	7.00E-67	256
Xa21_50416					Chr03	98.44	2.00E-61	238
Xa21_50441	chr12	96.23	7.00E-37	155				
Xa21_50497	chr12	98.68	4.00E-75	283				
Xa21_50546					Chr10	94.06	4.00E-36	153
Xa21_50725					Chr10	86.96	3.00E-07	58
Xa21_50734					Chr11	96.61	7.00E-16	85.7
Xa21_51047	chr06	95.05	2.00E-91	337				
Xa21_51086	chr07	82.5	1.00E-12	75.8				
Xa21_51106					Chrunknown	96.3	4.00E-96	353
Xa21_51120					Chr01	99.08	1.00E-114	414
Xa21_51359	chr02	92.31	1.00E-10	67.9				
Xa21_51411					Chr06	93.85	4.00E-19	97.6
Xa21_51572					Chr03	98.07	7.00E-104	379
Xa21_51622					Chr03	94.23	1.00E-37	159
Xa21_51654					Chr06	96.91	2.00E-98	361

Xa21_51681	chr01	98.25	9.00E-49	194				
Xa21_51704	chr06	99.22	3.00E-64	246				
Xa21_51707					Chr10	94.74	1.00E-07	60
Xa21_51875	chr09	94.09	5.00E-89	329				
Xa21_51892	chr11	96.88	3.00E-90	333				
Xa21_51998					Chr07	96.17	7.00E-73	276
Xa21_52004	chr01	84.81	2.00E-08	61.9				
Xa21_52032	chr03	99.55	7.00E-119	428				
Xa21_52060	chr08	99.16	1.00E-58	228				
Xa21_52089					Chr06	93.55	2.00E-49	198
Xa21_52144	chr01	100	5.00E-101	369				
Xa21_52284	chr01	99.13	2.00E-119	430				
Xa21_52425	chr01	97.44	7.00E-11	69.9				
Xa21_52441	chr01	89.33	3.00E-13	77.8				
Xa21_52545	chr02	99.08	3.00E-115	416				
Xa21_52553	chr11	97.32	7.00E-50	198				
Xa21_52571					Chrunknown	95.37	2.00E-42	174
Xa21_52640					Chrunknown	100	7.00E-70	266
Xa21_52738					Chrunknown	99.49	1.00E-102	375
Xa21_52827					Chrunknown	99.57	2.00E-116	420
Xa21_52897					Chr11	95.48	2.00E-76	287
Xa21_52957	chr12	96.67	1.00E-98	361				
Xa21_52979	chr12	86.84	2.00E-11	71.9				
Xa21_52991	chr09	86.62	2.00E-29	131				
Xa21_53007					Chr03	97.8	2.00E-39	165
Xa21_53078					Chr05	99.04	2.00E-49	198
Xa21_53170					Chrunknown	100	2.00E-129	464
Xa21_53171					Chr04	98.96	1.00E-99	365
Xa21_53213	chr11	94.44	9.00E-66	252				
Xa21_53280	chr03	97.19	3.00E-77	289				
Xa21_53317					Chr01	94.87	2.00E-08	61.9
Xa21_53451					Chrunknown	98.78	6.00E-83	309
Xa21_53656	chr08	99.16	1.00E-58	228				

Xa21_54025	chr03	100	4.00E-06	54				
Xa21_54040					Chr10	92	8.00E-14	79.8
Xa21_54208	chr01	95.24	1.00E-28	127				
Xa21_54369					Chr02	95.81	2.00E-70	268
Xa21_54473					Chrunknown	91.55	7.00E-18	93.7
Xa21_54532	chr03	99.07	2.00E-51	204				
Xa21_54539					Chrunknown	99.57	2.00E-126	454
Xa21_54787					Chr04	99.07	5.00E-111	402
Xa21_54796	chr12	98.91	4.00E-92	339				
Xa21_54897	chr11	80.65	7.00E-11	69.9				
Xa21_55011					Chr01	92.86	1.00E-36	155
Xa21_55093	chr06	91.87	8.00E-31	135				
Xa21_55111	chr06	98.32	4.00E-102	373				
Xa21_55131	chr10	89.04	5.00E-15	83.8				
Xa21_55268					Chr09	100	5.00E-34	147
Xa21_55316					Chr06	93.33	2.00E-09	65.9
Xa21_55456					Chrunknown	89.83	9.00E-11	69.9
Xa21_55678					Chrunknown	85.71	6.00E-09	63.9
Xa21_55987	chr12	86.41	6.00E-21	103				
Xa21_56060					Chrunknown	91.55	7.00E-18	93.7
Xa21_56097	chr02	98.82	2.00E-69	264				
Xa21_56125					Chrunknown	100	1.00E-117	424
Xa21_56195					Chr07	98.19	8.00E-110	398
Xa21_56273	chr07	96.06	6.00E-47	188				
Xa21_56285	chr11	97.73	2.00E-109	396				
Xa21_56442					Chr02	100	1.00E-102	375
Xa21_56457	chr01	95.07	5.00E-58	226				
Xa21_56507	chr08	97.36	2.00E-110	400				
Xa21_56667					Chr10	92.31	6.00E-06	54
Xa21_56836					Chr10	99.54	5.00E-114	412
Xa21_56991					Chrunknown	99.19	9.00E-62	238
Xa21_57041					Chrunknown	98.49	1.00E-99	365
Xa21_57102					Chr10	98.2	2.00E-110	400

Xa21_57115					Chrunknown	92.47	2.00E-67	258
Xa21_57156	chr03	96.33	9.00E-46	184				
Xa21_57178					Chr10	96.68	3.00E-94	347
Xa21_57206	chr09	89.9	4.00E-62	240				
Xa21_57219	chr09	97.38	3.00E-112	406				
Xa21_57278	chr06	82.46	6.00E-39	163				
Xa21_57300	chr01	89.33	3.00E-13	77.8				
Xa21_57369	chr12	100	2.00E-115	416				
Xa21_57379	chr03	99.41	5.00E-86	319				
Xa21_57428	chr06	96.62	3.00E-90	333				
Xa21_57515	chr11	97.56	5.00E-98	359				
Xa21_57530	chr08	93.81	2.00E-70	268				
Xa21_57720					Chr04	100	5.00E-59	230
Xa21_57816					Chr12	90.7	7.00E-06	54
Xa21_57878	chr03	95.45	3.00E-22	107				
Xa21_57934					Chr04	96.91	6.00E-73	276
Xa21_57988	chr03	96.46	2.00E-88	327				
Xa21_58084	chr01	99.01	9.00E-106	385				
Xa21_58101					Chrunknown	93.62	9.00E-11	69.9
Xa21_58120					Chrunknown	100	2.00E-132	474
Xa21_58211					Chr06	94.59	1.00E-56	222
Xa21_58301	chr08	97.16	4.00E-83	309				
Xa21_58307					Chr04	99.42	4.00E-90	333
Xa21_58417	chr01	97.99	9.00E-72	272				
Xa21_58494					Chr05	96.3	2.00E-113	410
Xa21_58732	chr06	96.43	8.00E-60	232				
Xa21_58774					Chr09	97.3	2.00E-44	180
Xa21_58814					Chr06	92.86	5.00E-77	289
Xa21_58819					Chr03	97.54	9.00E-104	379
Xa21_58888	chr01	89.66	1.00E-10	67.9				
Xa21_58919					Chr12	99.41	2.00E-72	274
Xa21_59052					Chrunknown	94.87	1.00E-08	61.9
Xa21_59111	chr11	100	4.00E-54	212				

Xa21_59120	chr04	91.87	1.00E-38	161				
Xa21_59131					Chr06	99.49	2.00E-104	381
Xa21_59177					Chr04	95.74	1.00E-08	61.9
Xa21_59289					Chr04	99.11	2.00E-54	214
Xa21_59302	chr06	95.1	7.00E-88	325				
Xa21_59303					Chr04	85.58	5.00E-40	167
Xa21_59328	chr06	98.31	1.00E-95	351				
Xa21_59503					Chr02	100	2.00E-119	430
Xa21_59541	chr01	99.1	4.00E-117	422				
Xa21_59600					Chr04	100	1.00E-17	91.7
Xa21_59687	chr09	99.54	1.00E-117	424				
Xa21_59711	chr06	92.69	2.00E-82	307				
Xa21_59739					Chr05	94.12	4.00E-21	103
Xa21_59770					Chr12	100	9.00E-62	238
Xa21_59838					Chrunknown	100	2.00E-107	391
Xa21_59927	chr05	99.05	2.00E-50	200				
Xa21_59945	chr02	100	2.00E-128	460				
Xa21_60018					Chrunknown	96	4.00E-90	333
Xa21_60165	chr11	97.25	1.00E-105	385				
Xa21_60218					Chr08	100	9.00E-45	182
Xa21_60329	chr11	88.46	1.00E-06	56				
Xa21_60330					Chr07	98.12	5.00E-105	383
Xa21_60355					Chrunknown	95.6	2.00E-79	297
Xa21_60446					Chr03	100	3.00E-29	131
Xa21_60501	chr02	97.67	2.00E-36	155				
Xa21_60512					Chr04	90.57	2.00E-09	65.9
Xa21_60535	chr03	98.4	5.00E-95	349				
Xa21_60554	chr01	95.85	1.00E-86	321				
Xa21_60571	chr12	100	4.00E-73	276				
Xa21_60760	chr02	100	2.00E-67	258				
Xa21_60888	chr07	100	2.00E-81	303				
Xa21_60967	chr08	94.44	3.00E-65	250				
Xa21_60973	chr12	90.35	1.00E-27	125				

Xa21_61128					Chrunknown	81.68	2.00E-08	61.9
Xa21_61148					Chr09	98.81	1.00E-133	478
Xa21_61326					Chr06	93.75	6.00E-86	319
Xa21_61420	chr09	94.05	4.00E-28	127				
Xa21_61570	chr07	98.86	3.00E-87	323				
Xa21_61630	chr03	87.43	6.00E-27	123				
Xa21_61635	chr01	97.57	5.00E-101	369				
Xa21_61669	chr04	92.7	3.00E-65	250				
Xa21_61865					Chrunknown	97.08	4.00E-109	396
Xa21_61875					Chr11	91.98	2.00E-76	287
Xa21_61996					Chr05	96	2.00E-98	361
Xa21_62068					Chr04	96.88	1.00E-23	111
Xa21_62090					Chr01	92.5	4.00E-90	333
Xa21_62213	chr09	98.27	3.00E-118	426				
Xa21_62243					Chrunknown	100	9.00E-62	238
Xa21_62272					Chr12	99.25	1.00E-64	248
Xa21_62339					Chr10	96.68	3.00E-94	347
Xa21_62472					Chr04	100	2.00E-83	311
Xa21_62602					Chr04	93.6	8.00E-98	359
Xa21_62681					Chrunknown	99.35	6.00E-77	289
Xa21_62710					Chrunknown	100	2.00E-06	56
Xa21_62800	chr06	94.64	2.00E-82	307				
Xa21_62874	chr02	90.54	5.00E-15	83.8				
Xa21_62900	chr03	98.4	5.00E-95	349				
Xa21_63148					Chr10	86.96	3.00E-07	58
Xa21_63254					Chr06	95.63	5.00E-68	260
Xa21_63327					Chr09	94.07	5.00E-96	353
Xa21_63337	chr12	100	3.00E-53	210				
Xa21_63415	chr11	94.55	1.00E-92	341				
Xa21_63431					Chr10	99.54	2.00E-116	420
Xa21_63603					Chr02	98.44	2.00E-61	238
Xa21_63632	chr11	97.52	5.00E-67	256				
Xa21_63636					Chr04	100	7.00E-70	266

Xa21_63711					Chr09	100	1.00E-121	438
Xa21_63885					Chrunknown	91.42	1.00E-78	295
Xa21_63912	chr07	97.02	7.00E-72	272				
Xa21_63968					Chr10	96.77	3.00E-09	65.9
Xa21_64058	chr01	95.87	4.00E-48	192				
Xa21_64102	chr02	97.87	1.00E-28	129				
Xa21_64115	chr06	96.22	1.00E-98	361				
Xa21_64171	chr06	84.17	3.00E-16	87.7				
Xa21_64178					Chr06	97.37	1.00E-89	331
Xa21_64361					Chr09	94.92	8.00E-18	93.7
Xa21_64396					Chr09	99.15	1.00E-124	448
Xa21_64419	chr11	94.2	1.00E-92	341				
Xa21_64427					Chr06	98.6	4.00E-96	353
Xa21_64466					Chr04	99.12	6.00E-120	432
Xa21_64739					Chr06	97.86	2.00E-79	297
Xa21_64811	chr03	95.28	2.00E-50	200				
Xa21_64854					Chr02	100	2.00E-132	474
Xa21_64872	chr06	99.12	3.00E-118	426				
Xa21_64928					Chr05	100	6.00E-46	186
Xa21_65008					Chrunknown	84.62	1.00E-07	60
Xa21_65013	chr05	100	2.00E-81	303				
Xa21_65020	chr02	98.21	2.00E-21	103				
Xa21_65064	chr01	98.52	1.00E-101	371				
Xa21_65132					Chr09	99.59	2.00E-132	474
Xa21_65238	chr10	92.59	2.00E-11	71.9				
Xa21_65326					Chrunknown	98.71	4.00E-75	283
Xa21_65447					Chr07	96.04	1.00E-93	345
Xa21_65479					Chrunknown	94.15	8.00E-73	276
Xa21_65480	chr01	100	6.00E-88	325				
Xa21_65563	chr01	99.31	6.00E-72	272				
Xa21_65677					Chr01	99.28	2.00E-70	268
Xa21_65759					Chr10	100	6.00E-70	266
Xa21_65787					Chr11	96.77	5.00E-06	54

Xa21_65830	chr10	100	2.00E-12	73.8				
Xa21_65933					Chr07	96.7	1.00E-99	365
Xa21_65947					Chrunknown	87.8	2.00E-12	75.8
Xa21_65995					Chrunknown	95.54	1.00E-55	218
Xa21_66208					Chrunknown	94.26	9.00E-82	305
Xa21_66215	chr08	91.95	1.00E-86	321				
Xa21_66346	chr02	100	5.00E-27	123				
Xa21_66440					Chr01	98.8	9.00E-82	305
Xa21_66457	chr07	100	1.00E-110	400				
Xa21_66528	chr08	100	1.00E-104	381				
Xa21_66620	chr12	97.61	3.00E-100	367				
Xa21_66678					Chr07	92.86	2.00E-28	127
Xa21_66748					Chr12	98.61	2.00E-30	135
Xa21_67001	chr11	92.92	1.00E-92	341				
Xa21_67012					Chr10	98.98	6.00E-46	186
Xa21_67043					Chr07	97.41	4.00E-93	343
Xa21_67111					Chr11	96.05	8.00E-104	379
Xa21_67186	chr06	96.88	6.00E-107	389				
Xa21_67203					Chr05	97.06	9.00E-08	60
Xa21_67409					Chr05	95.92	3.00E-85	317
Xa21_67430	chr06	97.78	3.00E-81	303				
Xa21_67462	chr07	81.65	3.00E-07	58				
Xa21_67540					Chr05	99.44	5.00E-93	343
Xa21_67676	chr12	90.95	5.00E-61	236				
Xa21_67859					Chrunknown	94.35	5.00E-33	143
Xa21_68061					Chr02	98.51	3.00E-103	377
Xa21_68095					Chr09	93.02	1.00E-22	109
Xa21_68106	chr02	92.31	2.00E-10	67.9				
Xa21_68112	chr04	99.01	8.00E-48	192				
Xa21_68158					Chr06	94.12	3.00E-54	214
Xa21_68303					Chrunknown	91.86	1.00E-24	115
Xa21_68317	chr05	85.23	4.00E-09	63.9				
Xa21_68355					Chr11	99.45	1.00E-96	355

Xa21_68473	chr07	98.99	5.00E-104	379				
Xa21_68496	chr11	100	2.00E-119	430				
Xa21_68612	chr11	97.51	7.00E-113	408				
Xa21_68964	chr01	98.58	9.00E-109	394				
Xa21_69047	chr08	98.41	2.00E-88	327				
Xa21_69130	chr03	88.19	4.00E-27	123				
Xa21_69225					Chr04	97.67	2.00E-129	464
Xa21_69279	chr09	93.55	4.00E-58	226				
Xa21_69384	chr08	99.16	1.00E-58	228				
Xa21_69394					Chr01	98.37	2.00E-58	228
Xa21_69466	chr04	97.35	2.00E-110	400				
Xa21_69489	chr12	98.78	6.00E-83	309				
Xa21_69512					Chrunknown	99.59	3.00E-134	480
Xa21_69573	chr02	92.16	6.00E-10	65.9				
Xa21_69824					Chr04	93.98	2.00E-76	287
Xa21_69995					Chr12	93.48	3.00E-19	97.6
Xa21_70010					Chr04	98.37	1.00E-74	281
Xa21_70047					Chrunknown	98.25	9.00E-110	398
Xa21_70064					Chr03	98.25	1.00E-111	404
Xa21_70065					Chr01	98.66	6.00E-114	412
Xa21_70111	chr01	97.13	7.00E-82	305				
Xa21_70287					Chr04	97.69	2.00E-83	311
Xa21_70592					Chr07	98.99	2.00E-46	188
Xa21_70809					Chr10	100	2.00E-113	410
Xa21_70855					Chr11	100	8.00E-88	325
Xa21_70896					Chr04	96.33	2.00E-38	161
Xa21_70936					Chrunknown	89.8	2.00E-24	115
Xa21_70982	chr02	91.95	4.00E-25	117				
Xa21_70984	chr08	90.76	7.00E-35	149				
Xa21_70992					Chrunknown	99.12	3.00E-40	167
Xa21_71004					Chr05	99.54	1.00E-117	424
Xa21_71044	chr08	90.91	8.00E-07	56				
Xa21_71093					Chr04	97.66	6.00E-57	222

Xa21_71137	chr01	100	2.00E-09	63.9				
Xa21_71340					Chr04	99.16	4.00E-127	456
Xa21_71381					Chr07	91.15	5.00E-31	137
Xa21_71442	chr07	99.48	2.00E-103	377				
Xa21_71447	chr08	99.12	1.00E-120	434				
Xa21_71535					Chr10	100	1.00E-59	232
Xa21_71537					Chr07	93.3	3.00E-81	303
Xa21_71682					Chr06	94.04	7.00E-52	206
Xa21_71704	chr07	100	2.00E-07	58				
Xa21_71916					Chr05	86.46	4.00E-16	87.7
Xa21_71950					Chr10	96.68	3.00E-94	347
Xa21_72159	chr04	100	2.00E-38	161				
Xa21_72161					Chrunknown	93.14	9.00E-18	93.7
Xa21_72166					Chr04	100	3.00E-60	234
Xa21_72175					Chr10	96.77	3.00E-09	65.9
Xa21_72192					Chr01	97.65	5.00E-105	383
Xa21_72204	chr06	99.49	1.00E-104	381				
Xa21_72220	chr06	98.95	2.00E-44	180				
Xa21_72239					Chrunknown	95.37	2.00E-42	174
Xa21_72284					Chr11	96.37	6.00E-86	319
Xa21_72291					Chr06	95.38	7.00E-26	119
Xa21_72455	chr07	99.04	5.00E-50	198				
Xa21_72497	chr06	97.06	4.00E-42	172				
Xa21_72581	chr06	100	1.00E-120	434				
Xa21_72730	chr01	99.08	1.00E-114	414				
Xa21_72739	chr07	99.5	2.00E-106	387				
Xa21_72754	chr01	97.84	2.00E-113	410				
Xa21_72848					Chr11	100	3.00E-109	396
Xa21_73002					Chrunknown	99.21	3.00E-63	244
Xa21_73012					Chr03	96.49	1.00E-77	291
Xa21_73193	chr05	95.12	1.00E-09	65.9				
Xa21_73501					Chr07	87.68	3.00E-51	204
Xa21_73567					Chr10	98.2	2.00E-110	400

Xa21_73588	chr04	99.43	6.00E-92	339				
Xa21_73604	chr04	94.29	5.00E-06	54				
Xa21_73614					Chrunknown	100	3.00E-34	147
Xa21_73640					Chr04	99.21	1.00E-62	242
Xa21_73666					Chr08	100	2.00E-119	430
Xa21_73834					Chr05	99.56	1.00E-121	438
Xa21_74063					Chr02	98.99	7.00E-101	369
Xa21_74078	chr12	98.25	8.00E-94	345				
Xa21_74148					Chrunknown	99.07	2.00E-111	404
Xa21_74237	chr10	98.52	1.00E-101	371				
Xa21_74271					Chr06	86.4	2.00E-43	178
Xa21_74352	chr08	98.66	2.00E-116	420				
Xa21_74384	chr08	95.08	3.00E-15	83.8				
Xa21_74453	chr07	97.94	2.00E-28	127				
Xa21_74529					Chrunknown	98.99	2.00E-101	371
Xa21_74612					Chr05	98.66	2.00E-104	381
Xa21_74724					Chr03	99.06	7.00E-107	389
Xa21_74770	chr08	100	9.00E-06	52				
Xa21_74972	chr05	99.56	2.00E-122	440				
Xa21_74978					Chrunknown	99.56	2.00E-119	430
Xa21_75278	chr06	92.74	4.00E-64	246				
Xa21_75424					Chr04	99.02	2.00E-107	391
Xa21_75450	chr10	100	2.00E-60	234				
Xa21_75459	chr01	99.21	1.00E-60	234				
Xa21_75475					Chrunknown	99.21	1.00E-60	234
Xa21_75529					Chrunknown	90.16	6.00E-55	216
Xa21_75576	chr02	92.31	2.00E-10	67.9				
Xa21_75728					Chr03	95.56	4.00E-12	73.8
Xa21_75790					Chr12	99.35	2.00E-79	297
Xa21_75795					Chr03	99.57	3.00E-125	450
Xa21_75905					Chr10	94.29	4.00E-06	54
Xa21_76009	chr03	93.75	4.00E-09	63.9				
Xa21_76010					Chrunknown	96.97	4.00E-74	280

Xa21_76015					Chr06	98.18	3.00E-109	396
Xa21_76105					Chr04	98.66	2.00E-116	420
Xa21_76214					Chrunknown	95.78	3.00E-91	337
Xa21_76229	chr01	99.01	2.00E-103	377				
Xa21_76284					Chr10	93.98	2.00E-61	238
Xa21_76304					Chrunknown	98.97	2.00E-101	371
Xa21_76363	chr06	100	5.00E-66	252				
Xa21_76462	chr06	87.13	6.00E-45	182				
Xa21_76718					Chr08	99.5	3.00E-106	387
Xa21_76754					Chr04	98.91	1.00E-92	341
Xa21_76756	chr01	96.39	2.00E-32	141				
Xa21_76799	chr10	98.68	9.00E-100	365				
Xa21_76837					Chr04	94.14	3.00E-85	317
Xa21_76870					Chrunknown	94.86	4.00E-90	333
Xa21_76931	chr11	99.5	5.00E-107	389				
Xa21_76937	chr01	98.96	5.00E-45	182				
Xa21_77121	chr06	97.52	7.00E-94	345				
Xa21_77253	chr04	82.98	7.00E-08	60				
Xa21_77425	chr07	91.49	2.00E-82	307				
Xa21_77432	chr07	98.8	2.00E-37	157				
Xa21_77532					Chr06	88.89	4.00E-06	54
Xa21_77605					Chr02	98.51	3.00E-103	377
Xa21_77613					Chr04	99.21	1.00E-62	242
Xa21_77709					Chr05	100	6.00E-35	149
Xa21_77753					Chr04	99.21	1.00E-62	242
Xa21_77796					Chrunknown	93.13	3.00E-44	180
Xa21_77900	chr06	100	1.00E-19	97.6				
Xa21_77985					Chr10	98.82	7.00E-86	319
Xa21_78004					Chrunknown	95.26	4.00E-93	343
Xa21_78291	chr11	98.48	2.00E-100	367				
Xa21_78314	chr06	98.11	2.00E-75	283				
Xa21_78453					Chrunknown	96.86	9.00E-85	315
Xa21_78485	chr08	95.44	1.00E-102	375				

Xa21_78559	chr03	90.05	1.00E-74	281				
Xa21_78597	chr06	86.45	7.00E-27	123				
Xa21_78732	chr04	98.15	3.00E-77	289				
Xa21_78820					Chr08	84.71	4.00E-07	58
Xa21_78946					Chr04	99.19	2.00E-61	238
Xa21_78993	chr06	100	7.00E-88	325				
Xa21_79090	chr06	89.86	2.00E-14	81.8				
Xa21_79132	chr01	89.8	3.00E-07	58				
Xa21_79141	chr10	87.5	4.00E-09	63.9				
Xa21_79213					Chr06	94.44	1.00E-06	56
Xa21_79217					Chrunknown	95.22	8.00E-101	369
Xa21_79280					Chr01	91.39	4.00E-47	190
Xa21_79336					Chr12	97.67	1.00E-80	301
Xa21_79344					Chr02	99.59	3.00E-134	480
Xa21_79372					Chrunknown	98.86	8.00E-85	315
Xa21_79377					Chrunknown	93.64	8.00E-39	163
Xa21_79401	chr11	100	1.00E-58	228				
Xa21_79410	chr01	98.19	8.00E-91	335				
Xa21_79415					Chr05	96.33	2.00E-79	297
Xa21_79459	chr01	95.02	5.00E-92	339				
Xa21_79473					Chr06	94.34	8.00E-39	163
Xa21_79508					Chr11	98.54	1.00E-105	385
Xa21_79536					Chr03	97.65	6.00E-36	153
Xa21_79556	chr03	95.7	1.00E-31	137				
Xa21_79563					Chrunknown	91.55	7.00E-18	93.7
Xa21_79638					Chrunknown	83.7	1.00E-06	56
Xa21_79660					Chrunknown	100	2.00E-119	430
Xa21_79728					Chr04	97.67	5.00E-130	466
Xa21_79802	chr08	92.65	2.00E-69	264				
Xa21_79830					Chr10	96.7	8.00E-95	349
Xa21_79984	chr05	96.21	4.00E-52	206				
Xa21_80172					Chr06	94.47	9.00E-51	202
Xa21_80209					Chr05	96.08	2.00E-34	147

Xa21_80363					Chrunknown	97.47	3.00E-128	460
Xa21_80486	chr03	100	2.00E-91	337				
Xa21_80517					Chrunknown	98.18	3.00E-109	396
Xa21_80611					Chr05	98.99	1.00E-46	188
Xa21_80871	chr12	90.23	7.00E-73	276				
Xa21_80878					Chrunknown	96.27	3.00E-51	204
Xa21_81111	chr02	100	6.00E-53	208				
Xa21_81195					Chrunknown	96.15	1.00E-38	161
Xa21_81239	chr10	98.71	1.00E-77	291				
Xa21_81274					Chr01	99.46	7.00E-98	359
Xa21_81456	chr11	98.54	2.00E-106	387				
Xa21_81488					Chr05	100	2.00E-122	440
Xa21_81723	chr06	91.01	3.00E-50	200				
Xa21_81841	chr07	98.31	3.00E-23	109				
Xa21_81876	chr06	89.56	2.00E-48	194				
Xa21_82168	chr06	98.61	6.00E-110	398				
Xa21_82274	chr08	98.57	4.00E-108	392				
Xa21_82395	chr07	96.77	1.00E-54	214				
Xa21_82435					Chrunknown	96.88	2.00E-06	56
Xa21_82454	chr02	91.95	4.00E-25	117				
Xa21_82504					Chr02	100	4.00E-51	202
Xa21_82574					Chrunknown	92	2.00E-10	67.9
Xa21_82604	chr07	96.86	6.00E-88	325				
Xa21_82725	chr10	85.44	1.00E-15	85.7				
Xa21_82767	chr11	94.39	5.00E-89	329				
Xa21_82788	chr12	95.13	1.00E-98	361				
