# Supplementary material for: Global characterization of the root transcriptome of a wild species of rice, Oryza longistaminata, by deep sequencing
Source: BMC Genomics. 2010 Dec 15;11:705. doi: 10.1186/1471-2164-11-705 (PMC3016420; doi:10.1186/1471-2164-11-705)
Supplement: Additional file 5 — O. longistaminata ESTs analyzed by RT-PCR from cDNA of pot-grown O. longistaminata rice roots. [file 1471-2164-11-705-S5.PDF]

Additional file 5:

Table S4: 19 novel *O. longistaminata* ESTs analyzed by RT-PCR from cDNA of pot-grown *O. longistaminata* rice roots.

ESTs	Forward primers (5'-3')	Reverse primer (5'-3')	Amplification size (bp)[a]	RT-PCR[b]
Xa21_2330	TGTACTGTGGGGGAGGTTGT	GGTCAGCAGCAACCAAAAAT	288	
Xa21_3241	CCAAATTCCCTGAGTTGGAA	CGTCCTTTGCATTCATCAGA	236	+
Xa21_5950	GTTGGACTGTTTGGCTTGGT	GACTCGAGAACCGAAACGAG	205	
Xa21_8650	GCAGTGTCCTGTGTGCAAGT	TCGAATCGTAGCATCACAGC	287	
Xa21_11369	AACGACTCTGTTGCCTCCTG	ACTGAACCAGAAGCCTCCAA	263	
Xa21_11949	TCGACTTGAGCGCCTTTTAT	GGTTGTCTTGCAACGCAGTA	207	+
Xa21_14234	GCGAGTCACCTTCTTTGCTT	CCACAAAACGGAGGATTCAC	223	+
Xa21_25173	TCTTTTTGGATTGGCTGGAG	GCCCACAAAGATACGAGGAA	197	+
Xa21_26573	GCGAATTCCCGATACTTTGA	GCAGACGCCTAACCTTCAAC	271	+
Xa21_26636	TGGAGATGCATTCCGTATGA	ACTTCAGGTCCGACAACACC	258	
Xa21_37257	TGATGGGTGCTCCTACAACA	CAACCGTCCAGCTAACGAAT	273	
Xa21_38071	AATCATACCGGCATTTGCTC	CTCGATTCAAGAGCGGAAAC	170	
Xa21_38604	AACTCGTACAAAAAGCCTCACA	TTTTCGGCATCTTTCTTTGC	172	
Xa21_42295	TTGGACCTACCAGACCAACC	GAGGCGTTGACAGAGAAAGC	154	
Xa21_43033	GCTGCTTTTGCAAATCAACA	AAACATGGGTGTTGGTGGAT	201	
Xa21_43245	GCCCTCTCTGGATACCCTTC	GCACGCATCAAATGAGAAGA	170	
Xa21_57504	GGTTGGTATGCGTCATGTCTT	ATTGCAGATGAACCCTCGAT	249	+
Xa21_62699	TTTTGATTTCCGTTTCCATTG	ACGCGGAATCAAGACAATTT	188	
Xa21_81623	GCGAAGGTGTTGGTACTTCC	TTGAAATGTGAATCGGCTGT	240	

[a] Expected and /or observed size of the DNA fragment after amplification by RT-PCR.

[b] "+" indicates an amplification product obtained from cDNAs of pot-grown *O. longistaminata* rice roots.
